# Comparison of One-Year Outcome of Intravitreal Aflibercept with or without Photodynamic Therapy for Polypoidal Choroidal Vasculopathy

**DOI:** 10.3390/medicina60081311

**Published:** 2024-08-14

**Authors:** Hsin-Yu Weng, Fang-Ting Chen, Ling-Uei Wang, Tzu-Lun Huang, Wei-Ting Ho, Pei-Yao Chang, Yung-Ray Hsu, Yun-Ju Chen, Jia-Kang Wang

**Affiliations:** 1 Department of Ophthalmology, Far Eastern Memorial Hospital, New Taipei City 220, Taiwan; fiona700816@gmail.com (F.-T.C.); huang.tzulum@gmail.com (T.-L.H.); rbaggioh@gmail.com (W.-T.H.); peiyao@seed.net.tw (P.-Y.C.); scherzoray@gmail.com (Y.-R.H.); yjchen2006@ntu.edu.tw (Y.-J.C.); 2 Department of Medicine, National Taiwan University, Taipei City 100, Taiwan; 3 Department of Electrical Engineering, Yuan Ze University, Taoyuan City 320, Taiwan; 4 Department of Medicine, National Yang Ming University, Taipei City 112, Taiwan

**Keywords:** intravitreal injection, aflibercept, photodynamic therapy, verteporfin, polypoidal choroidal vasculopathy

## Abstract

*Background and Objectives*: Our study compared the visual and anatomical outcomes of polypoidal choroidal vasculopathy (PCV) patients receiving intravitreal aflibercept (IVA) with or without photodynamic therapy (PDT) over 12 months. *Materials and Methods*: This retrospective study was performed for 60 eyes from 60 patients with treatment-naïve PCV. Thirty eyes were treated using IVA monotherapy (IVA group), and thirty eyes were treated using a combination of IVA with PDT (IVA/PDT group). The baseline characteristics, treatment outcomes, and retreatment rates were compared between the two groups over a one-year follow-up period. *Results*: The best-corrected visual acuity (BCVA) was found to have improved significantly in the IVA/PDT group at every 3-month visit. However, no significant BCVA improvement was observed in the IVA group. A significantly lower retreatment rate and higher dry macula rate were found in the IVA/PDT group than that in the IVA group. In the entire population of the study, a better baseline vision and younger age were associated with better final visual outcomes. Retreatment was associated with poor baseline BCVA and IVA monotherapy. *Conclusions*: The combination of IVA and PDT may offer superior visual improvement and a higher dry macula rate compared to IVA monotherapy in the treatment of PCV patients while requiring fewer retreatments over 12 months.

## 1. Introduction

Polypoidal choroidal vasculopathy (PCV) is considered a subtype or variant of neovascular age-related macular degeneration (AMD). A typical characteristic of PCV is the presence of polypoidal choroidal lesions with or without a branching vascular network, detected using indocyanine green angiography (ICGA) [1,2,3]. PCV is more prevalent in the Asian population than in the white population. PCV accounts for 23.0–54.7% of neovascular AMD patients in Asians and 8–13% of Caucasians [4,5,6,7].

The natural course of the disease is variable. Half of the patients have favorable visual outcomes, while others suffer from macular atrophy and vision loss [8,9]. The treatment options for PCV include the intravitreal injection (IVI) of anti-vascular endothelial growth factor (anti-VEGF) agents, photodynamic therapy (PDT), or a combination of both.

The EVEREST study found that the complete polyp regression rates in combination therapy of PDT and intravitreal ranibizumab (IVR) or in PDT alone were higher than in IVR monotherapy [10]. The EVEREST II study reported that combined PDT and IVR achieved better visual outcomes, more polyp regression, and fewer treatment times compared with IVR monotherapy [11].

Aflibercept is an anti-VEGF agent that is more potent and has a longer half-life than ranibizumab or bevacizumab [12]. Intravitreal aflibercept (IVA) monotherapy or combined PDT and IVA have been reported as effective treatments for PCV [13,14], but studies involving a head-to-head comparison between the two treatment modalities are scant. Our study aims to compare the effect of IVA with or without PDT for patients with PCV over 12 months and to evaluate the clinical factors associated with visual outcomes and the rate of retreatment.

## 2. Materials and Methods

This retrospective medical chart review study was conducted at the Far Eastern Memorial Hospital in Taiwan from October 2015 to July 2020. The study was approved by the Institutional Review Board of Far Eastern Memorial Hospital (ethical approval office reference number: FEMH-107160-E) and followed the tenets of the Declaration of Helsinki. Sixty eyes from sixty patients with treatment-naïve PCV were enrolled in this study. A total of 30 patients received IVA monotherapy (IVA group), and the other 30 patients received a combination of IVA and PDT (IVA/PDT group). Prior to the treatment, written informed consent was secured from all participants. Patients with diabetic retinopathy, retinal vessel occlusion, rhegmatogenous retinal detachment, uveitis, or glaucoma were excluded from the study.

Prior to starting the treatments, all patients received detailed ophthalmic examinations, including best-corrected visual acuity (BCVA) measurements using the Snellen E chart, intraocular pressure, slit-lamp biomicroscopy, color fundus photography, spectral-domain optical coherence tomography (OCT) (RTVue; Optovue Inc., Fremont, CA, USA), and fundus fluorescein and indocyanine green angiography (FA/ICGA) (HRA; Heidelberg Engineering, Heidelberg, Germany). A fundus examination showed a subretinal exudate with or without hemorrhage. OCT revealed subretinal fluid and pigment epithelial detachment with or without hemorrhage. Polypoidal choroidal lesions in ICGA confirmed the diagnosis of PCV. Central retinal thickness (CRT) was defined as the vertical distance between the RPE and the inner-limiting membrane at the fovea, as measured using SD-OCT.

In the IVA group, the patients received three monthly injections of aflibercept (2.0 mg in 0.05 mL). In the IVA/PDT group, the patients received a combination therapy involving a standard PDT and a single IVA in a week. The standard PDT was performed according to the protocol used in the Treatment of Age-Related Macular Degeneration with Photodynamic Therapy studies [15,16]. After the initial treatment, all subjects were followed up monthly with examinations, including BCVA, slit-lamp biomicroscopy, a fundus examination, and OCT. The follow-up period was 12 months. FA/ICGA images were repeated 3 months after the initial treatment to detect the angiographic regression of polypoidal vascular lesions. Complete polyp regression was defined as no polypoidal lesion in repeated ICGA. Partial polyp regression was defined as a residual polypoidal lesion in repeated ICGA.

Retreatment was indicated in patients with recurrent or persistent subretinal or intraretinal fluid at the macula or a newly developed retinal hemorrhage. In the IVA group, retreatment only included IVA monotherapy. In the IVA/PDT group, IVA monotherapy was administered in patients with complete polyp regression, and combination therapy was administered in cases with no polyp regression or partial polyp regression. The retreatment rate was determined by the number of subjects in each group who received additional treatment. Both the number and rate of retreatments were documented.

The study outcomes were BCVA, CRT, complete polyp regression rate, and dry macula rate. The complete polyp regression rate was measured as the number of patients with complete polyp regression over the total number of patients in each group. The dry macular rate was measured as the number of patients with dry macula over the total number of patients. For statistical analysis, the BCVA measurements from the Snellen E chart were transformed into the logarithm of the minimum angle of resolution (LogMAR). The number of subjects who had significant visual change, i.e., BCVA gain or loss ≥ 0.3 LogMAR units from the baseline to final visits, was recorded. A dry macula was defined as no subretinal or intraretinal fluid at the macula detected via OCT. Additionally, the retreatment number and rate were compared between the groups.

The baseline characteristics and study outcomes were compared between the two treatment groups. The final BCVA and retreatment rates were evaluated for their association with relevant clinical factors. The paired t-test was used to compare the BCVA and CRT before and after treatment within a group. The independent t-test was used to compare the differences in the continuous variables between the groups. The chi-square test was used to evaluate the differences in the categorical variables between the groups. Pearson’s correlation coefficient was used to investigate the correlations among the clinical factors and treatment outcomes. Statistical analysis was performed using SPSS (v. 20.0; IBM, Armonk, NY, USA). A *p*-value less than 0.05 was considered statistically significant.

## 3. Results

### 3.1. Baseline Characteristics

A comparison of the baseline characteristic data of the two treatment groups is shown in Table 1. There were no significant differences in age, sex, lens status, baseline BCVA, or baseline CRT between the two groups.

### 3.2. Visual Acuity

**Figure 1:**
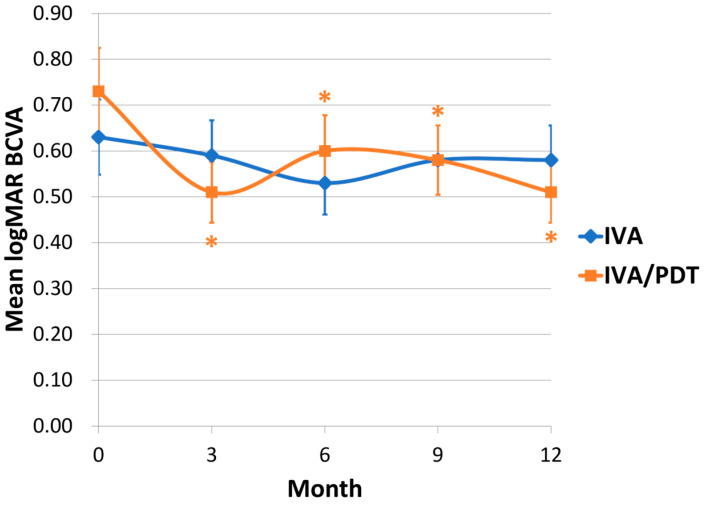
Changes in mean best-corrected visual acuity (BCVA) in LogMAR for two groups (* *p* < 0.05 compared with baseline BCVA).

The overall functional and anatomical outcomes are summarized in Table 2. The changes in the BCVA of the two groups are shown in Figure 1. In the IVA group, the mean BCVA in LogMAR changed from 0.63 ± 0.48 at the baseline to 0.58 ± 0.61 at 12 months. There was no significant improvement in the BCVA compared with the baseline at each 3-month visit (*p* = 0.561, 0.128, 0.575, and 0.608 at 3 months, 6 months, 9 months, and 12 months, respectively).

In the IVA/PDT group, the mean BCVA in LogMAR improved from 0.73 ± 0.65 at the baseline to 0.51 ± 0.59 at 12 months. The BCVA improved significantly at every 3-month visit (*p* = 0.002, 0.045, 0.045, and 0.010 at 3 months, 6 months, 9 months, and 12 months, respectively). No significant differences were observed in either the mean LogMAR BCVA or the change in BCVA from the baseline between the two groups at each 3-month follow-up visit.

### 3.3. Visual Gain or Visual Loss

At 12-month follow-up, 30.0% of patients in the IVA group and 33.3% of patients in the IVA/PDT group had a BCVA gain ≥ 0.3 LogMAR. BCVA loss ≥ 0.3 LogMAR occurred in 20.0% and 6.7% of patients in the IVA and IVA/PDT groups, respectively. Regarding the proportion of cases with visual gain or the loss of more than 0.3 LogMAR, no significant difference was found between the two groups (Table 2).

### 3.4. Central Retinal Thickness

**Figure 2:**
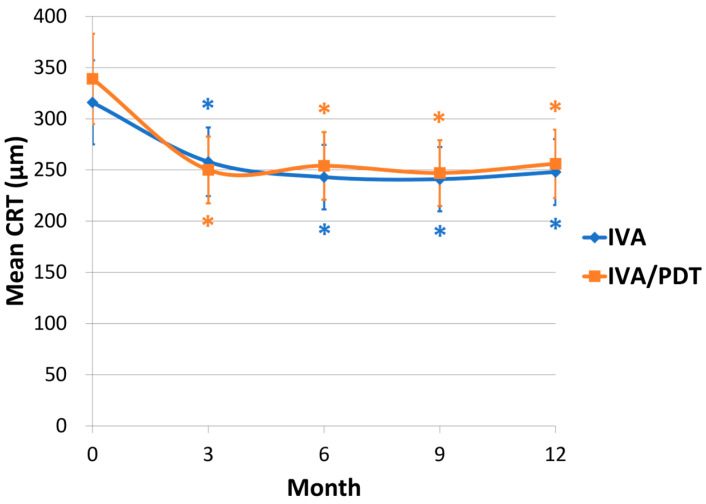
Changes in mean central retinal thickness (CRT) for two groups (* *p* < 0.05 compared with baseline CRT).

The changes in the CRT of the two groups are shown in Figure 2. The mean CRT reduced from 316 ± 129 μm at the baseline to 247 ± 44 μm at 12 months in the IVA group and from 339 ± 96 μm at the baseline to 256 ± 47 μm at 12 months in the IVA/PDT group. The CRT reduction compared to the baseline was significant at every 3-month follow-up in both groups. No significant differences were observed in either the mean CRT or the change in CRT from baseline between the two groups at each 3-month follow-up visit.

### 3.5. Polyp Regression Rate

**Figure 3:**
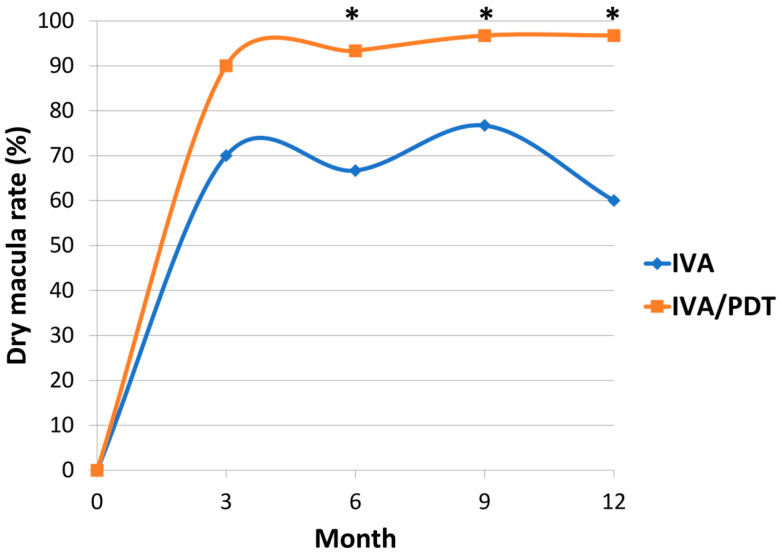
Changes in dry macula rate of two groups (* *p* < 0.05 compared between the two groups).

**Figure 4:**
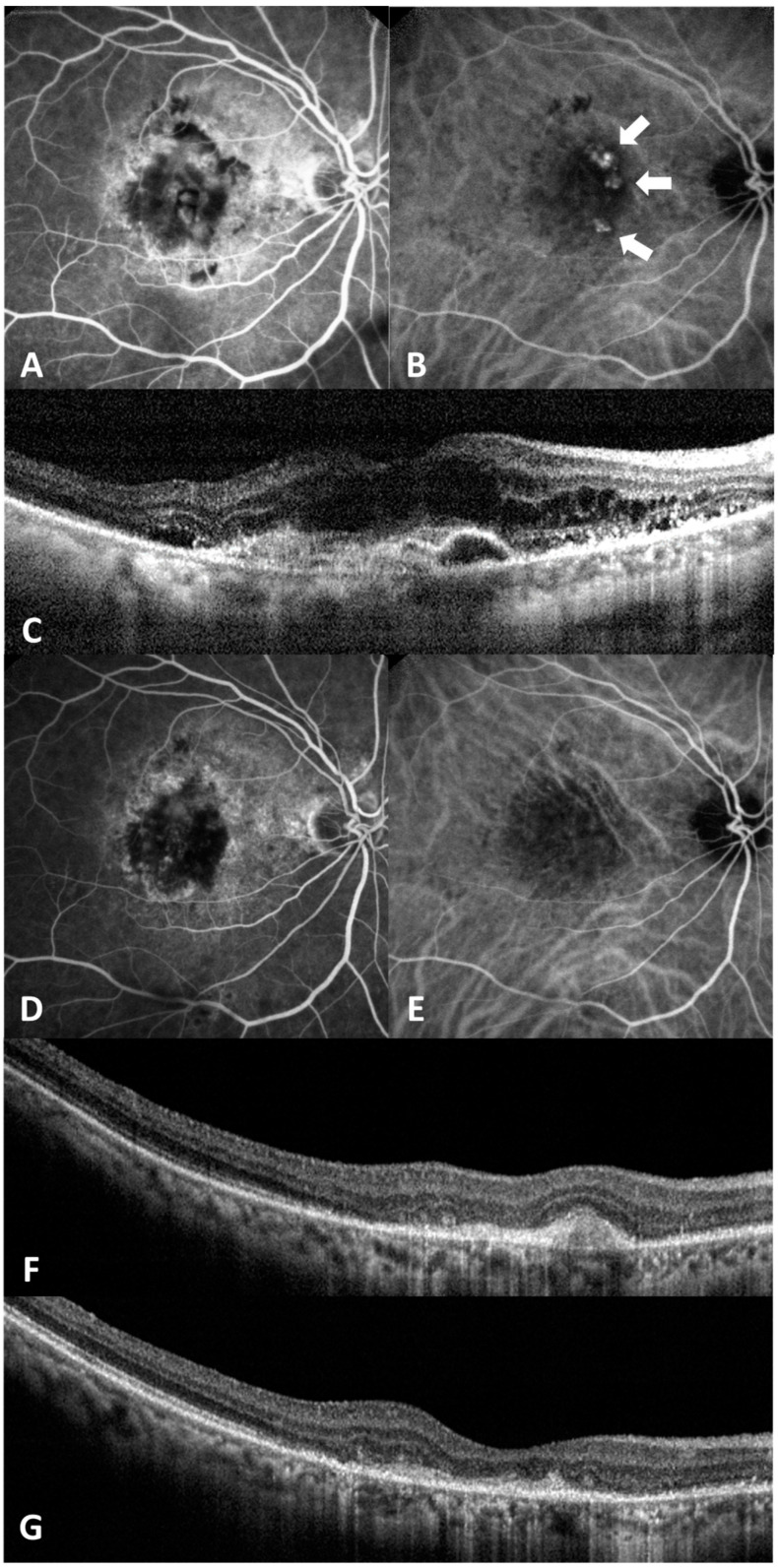
This is a 76-year-old man diagnosed with polypoidal choroidal vasculopathy (PCV) in his right eye. (**A**–**C**) are images taken prior to treatment, (**D**–**F**) are images captured 3 months after IVA/PDT treatment, and (**G**) is an image obtained 12 months after IVA/PDT treatment. (**A**) Fluorescein angiography (FA) shows fluorescein leakages. (**B**) Indocyanine green angiography (ICGA) shows polypoidal lesions (arrows). (**C**) Optical coherence tomography (OCT) reveals pigment epithelial detachment (PED), subretinal and intraretinal fluid. (**D**) FA shows staining without active leakage. (**E**) ICGA demonstrates complete regression of the polyp. (**F**,**G**) OCT reveals no subretinal or intraretinal fluid, although pigment epithelial detachment (PED) persists.

The complete polyp regression rate at 3 months was 50.0% and 53.3% in the IVA and IVA/PDT groups, respectively. There was no significant difference in the polyp regression rate between the two groups (*p* = 0.500) (Table 2). In the IVA group, the dry macula rate was 70.0% at 3 months, 66.7% at 6 months, 76.7% at 9 months, and 60.0% at 12 months. In the IVA/PDT group, the dry macula rate was 90.0% at 3 months, 93.3% at 6 months, 96.7% at 9 months, and 96.7% at 12 months. Between the two groups, the dry macula rate significantly differed at 6 months, 9 months, and 12 months (Figure 3). Figure 4 shows representative images of a case.

### 3.6. Retreatment

During the 12-month follow-up period, 17 patients (56.7%) from the IVA group received additional IVA monotherapy. In the IVA/PDT group, only six patients (20.0%) received additional combination therapy, and none received additional IVA monotherapy. The mean additional treatment number was 1.27 ± 1.31 in the IVA group and 0.20 ± 0.41 in the IVA/PDT group. There were significant differences in the retreatment rate and mean additional treatment number between the two groups (*p* = 0.004 and <0.001, respectively) (Table 3).

### 3.7. Associated Factors

Better visual outcome was associated with better baseline BCVA (r = 0.680, *p* < 0.001) and younger age (r = 0.366, *p* = 0.004) in all patients. There was no significant correlation between visual outcome with sex, lens status, the modality of treatment, or baseline CRT.

Retreatment was associated with poor-baseline BCVA (*p* = 0.017) and IVA monotherapy (*p* = 0.004). There was no correlation between retreatment with age, sex, lens status, or baseline CRT. Patients receiving retreatment had poor final BCVA (*p* = 0.005). Complete polyp regression at 3 months was associated with better final BCVA (*p* = 0.009), less final CRT (*p* = 0.044), and less retreatment (*p* < 0.001).

## 4. Discussion

This study compared the visual outcome, anatomical outcome, and retreatment of IVA with or without PDT for treating patients with PCV during a one-year study period. In our study, a significant improvement in BCVA was observed in the IVA/PDT group but not in the IVA group. However, no significant differences in mean LogMAR BCVA or change in BCVA were observed between the groups. Takayama K et al., Kikushima W et al., and Silva et al. also reported comparable visual outcomes in both IVA and IVA/PDT groups for PCV [17,18]. These prior studies discovered significant BCVA improvements in both groups, which were not exactly similar compared to the visual results of our study. A possible explanation for this is that the number of aflibercept intravitreal injections that can be administered under the National Health Insurance of Taiwan is limited to seven. In addition to the initial three loading doses, a maximum of four further IVA retreatments are permitted. This fact probably restricted visual improvement in the IVA group.

In the present study, CRT decreased significantly and was then maintained at 12 months in both groups; moreover, there were no significant differences in mean CRT or changes in CRT between the groups. Similarly, previous studies reported a significant reduction in CRT and subfoveal choroidal thickness in either the IVA group or the IVA/PDT group, and there were no significant intergroup differences [17,18]. Although our study observed no differences in CRT between the groups, the patients in the IVA group required more retreatment episodes but had lower dry macula rates. Recurrent and persistent disease activity led to damage to the macular structure, resulting in poor visual outcomes. However, the recurrent or persistent subretinal fluid was usually shallow, so CRT did not differ between the groups.

The IVA/PDT group had a significantly higher dry macula rate compared to the IVA group at the 6-month, 9-month, and 12-month follow-up in our study. The dry macula rates at 12 months in the IVA and IVA/PDT groups were 60.0% and 96.7%, respectively. Takayama K et al. and Silva R et al. found no significant difference in dry macula rate between the IVA and IVA/PDT groups [17]. The different treatment design was perhaps the reason for these diverse outcomes. Single PDT was performed along with three monthly loading aflibercept injections in the combination treatment in the previous two studies [17,19]. Additional PDT was permitted in the IVA/PDT group in this study, possibly providing better control to inhibit macular exudation from PCV.

The complete polyp regression rate at the 3-month follow-up was 50.0% and 53.3% in the IVA and IVA/PDT groups, respectively. No significant difference in the polyp regression rate was observed between the groups in our study. The complete polyp regression rate in a study conducted by Kikushima W et al. was 56.7% in the IVA group and 87.5% in the IVA/PDT group at 3 months, and 60.0% in the IVA group and 68.8% in the IVA/PDT group at 12 months. This rate was significantly different at 3 months but not significant at 12 months between the two groups [18]. Likewise, Silva et al. demonstrated comparable complete polyp occlusion between the two groups [19].

In the present study, fewer retreatments and additional treatments were required in the IVA/PDT group compared to the IVA group. Kikushima W et al. reported a significantly higher retreatment rate (63.6%) in the IVA group than in the IVA/PDT group (30.3%). Significantly, a greater number of additional IVA injections were administered in the IVA group, with a mean of 1.6 times compared to a mean of 0.42 times in the IVA/PDT group [18]. Takayama K et al. also reported that fewer IVA injections were required in the combination group [17].

PDT achieves favorable visual outcomes and high polyp regression rates in the treatment of PCV [10]. However, PDT induces hypoxia of the retinal pigment epithelium, which can stimulate VEGF production and subsequent angiogenesis [20]. The combination of PDT and anti-VEGF has synergistic effects on visual and anatomical outcomes [10,11]. Anti-VEGFs, such as bevacizumab, ranibizumab, and aflibercept, are reported to be effective in combination therapy for PCV [21]. The LAPTOP study compared IVR and PDT for the treating PCV and reported that the visual outcomes were better in the IVR group compared to the PDT group at both 12 months and 24 months after the treatment [22,23]. Although several studies have demonstrated the efficacy of combination therapy, the PLANET study, which evaluated IVA with or without rescue PDT in patients with PCV, reported that rescue PDT did not provide additional benefits [24].

Long-term outcomes and safety of combination therapy have also been documented. Liu S et al. reported favorable 3-year visual outcomes from IVR or IVA combined with PDT for PCV. Additionally, in patients who are refractory to IVR monotherapy, the combination with PDT was found to be more effective than switching to IVA [25]. Wataru K. et al. also reported good visual outcomes from combined IVR or IVA with PDT for PCV at 5-year follow-up [26]. Lee J. et al. reported 7-year outcomes of combined intravitreal bevacizumab (IVB) or IVR with PDT for PCV. In this study, the final visual outcomes were better in the group with polyp regression compared to the group with persistent polyp according to whether the polyps had regressed or not at 1-year follow-up [27]. In our study, complete polyp regression at 3 months was associated with better visual and anatomical outcomes and less retreatment.

We also demonstrated that the risk factors of poorer visual outcomes were worse BCVA at the baseline and older age. The patients with poor baseline BCVA and/or undergoing aflibercept monotherapy had a higher chance of repeated treatment.

Some limitations must be acknowledged in this study. First, this study was retrospective in nature. Second, the sample size was relatively small. Third, the duration of the follow-up was relatively short, and fourth, we did not record the polyp regression rate at each follow-up.

## 5. Conclusions

The combination of IVA and PDT may offer superior visual improvement and a higher dry macula rate compared to IVA monotherapy in the treatment of PCV patients while requiring fewer retreatments over 12 months. A better baseline vision and younger age were associated with better final visual outcomes, while poor baseline BCVA and IVA monotherapy were associated with a higher rate of retreatment.

## Figures and Tables

**Table 1:** Baseline characteristics of patients in the two groups.

	IVA (*n* = 30)	IVA/PDT (*n* = 30)	*p*-Value
Age (year)	65.0 ± 14.8	65.5 ± 12.8	0.896
Sex (male %)	53.3%	76.7%	0.052
Lens status (phakic %)	70.0%	66.7%	0.500
Baseline BCVA (LogMAR)	0.63 ± 0.48	0.73 ± 0.65	0.499
Baseline CRT (μm)	316 ± 129	339 ± 96	0.443

IVA: intravitreal aflibercept; PDT: photodynamic therapy; BCVA: best-corrected visual acuity; LogMAR: logarithm of the minimum angle of resolution; and CRT: central retinal thickness; *p* < 0.05 was considered statistically significant.

**Table 2:** Comparison of outcomes between the two groups.

	IVA (*n* = 30)	IVA/PDT (*n* = 30)	*p*-Value
Changes in BCVA from baseline to 12-month (LogMAR)	0.05 ± 0.48	0.22 ± 0.44	0.146
BCVA gain ≧ 0.3 LogMAR at 12-month	30.0%	33.3%	0.500
BCVA loss ≧ 0.3 LogMAR at 12-month	20.0%	6.7%	0.127
Changes in CRT from baseline to 12-month (μm)	69 ± 141	83 ± 98	0.642
3-month complete polyp regression rate	50.0%	53.3%	0.500
12-month dry macular rate	60.0%	96.7%	0.001 *

IVA: intravitreal aflibercept; PDT: photodynamic therapy; BCVA: best-corrected visual acuity; LogMAR: logarithm of the minimum angle of resolution; and CRT: central retinal thickness. * *p* < 0.05 was considered statistically significant.

**Table 3:** Retreatment comparison between the two groups.

	IVA (*n* = 30)	IVA/PDT (*n* = 30)	*p*-Value
Retreatment times			
1	4	6	
2	6	0	
3	6	0	
4	1	0	
Total (%)	17 (56.7%)	6 (20%)	0.004 *
Mean retreatment times	1.27 ± 1.31	0.20 ± 0.41	<0.001 *
Retreatment modality			
IVA only	17	0	
IVA/PDT combination	0	6	

IVA: intravitreal aflibercept; PDT: photodynamic therapy; * *p* < 0.05 was considered statistically significant.

## Data Availability

The original data presented in the study are openly available in the Supplementary Material files.

## Supplementary Materials

The following supporting information can be downloaded at https://www.mdpi.com/article/10.3390/medicina60081311/s1, Table S1: Raw data.
